# Prescriptions and Dispensing

**Published:** 1908-05-23

**Authors:** 


### Therapeutics.

### PRESCRIPTIONS AND DISPENSING.

PILLS (*continued*).

We were considering aloes in our last article, and have only a few points to add upon it. If the pill does not contain any fibrous material, such as a powdered vegetable drug, it is best to add a little liquorice. An excipient which is much used for making pills containing aloes is decoctum aloes compositum; a very little of this is effective, probably because of the potassium carbonate it contains. And although it is always undesirable to allow the use as excipient of anything that is itself a medicine, it is justified when, as here, the active ingredient is the same as that in the pill; besides, the quantity used is so small. Decoction of aloes should not be used in any case in which the potassium carbonate would be incompatible with some other ingredient of the pill.

Camphor is often a troublesome ingredient. It should in all cases be finely powdered. This can easily be done by adding a few drops of spirit to the camphor, and then rubbing it in a mortar. Tragacanth in some form is usually necessary. If camphor is the only ingredient, glycerin of tragacanth is best; in other cases powdered gum tragacanth and syrup of glucose may be better; it depends on the nature of the other ingredients. A few examples are:—

| | |
|---|---|
| ℞ Hydrargyri Subchloridi ... ... | gr. xij. |
| Camphoræ ... ... ... ... | gr. xij. |
| Asafœtidæ ... ... ... ... | gr. xij. |
| Pulveris Piperis Nigri ... ... | gr. xij. |
| Extracti Opii ... ... ... ... | gr. vj. |

Misce. Divide in pilulas xij.

Powder the camphor finely and mix with the calomel, the pepper, and 2 grains of powdered tragacanth; then work the extract of opium in with this; soften the asafœtida with a few drops of spirit, and then work all together into a mass.

Soap and oil are sometimes employed with camphor, as in the next prescription:—

| | |
|---|---|
| ℞ Camphoræ ... ... ... ... | gr. xxiv. |
| Olei Ricini ... ... ... ... | guttas iij. |
| Saponis ... ... ... ... | gr. ij. |

Fiant pilulæ xij.

The addition of a few drops of spirit makes this much easier to dispense.

Quinine and camphor are sometimes given together in pill form, as in the following prescription:—

| | |
|---|---|
| ℞ Quininæ Sulphatis ... ... ... | gr. j. |
| Camphoræ ... ... ... ... ... | gr. j. |

Fiat pilula. Mitte xx.

The camphor should be finely powdered, then thoroughly mixed with the quinine sulphate and 3 grains of powdered tragacanth, enough syrup being added for the massing.

Carbolic acid, more correctly known as phenol, is not infrequently prescribed in pill form, and it is apt to give some trouble. There are several ways of dealing with it, and the particular excipient to be employed in a given case will depend to some extent on what other ingredients are in the prescription. Powdered marsh-mallow root, with a small quantity of gum acacia, may be employed, and the mass made with syrup. Perhaps the excipient in most favour is powdered soap; if this be used, only neutral soap is permissible, as an alkali will combine with the phenol. It has been objected that even a neutral soap may react with the phenol and partly combine with it, but this is not likely to occur to any serious extent. The quantity of soap may be from ⅓ to 1 grain for each grain of phenol. If no powdered vegetable drug is in the prescription, a little liquorice will be an advantage; in some cases a little gum tragacanth and syrup will also be necessary.

Potassium permanganate, often used in pill form in gynæcological cases, is likely to give very serious trouble to anyone who omits to consider its powerful oxidising property; but it is easily made into pills when once the correct method is known. The ordinary excipients, such as confection of roses, glycerin of tragacanth, and so forth are inadmissible not only because they exercise a reducing action on the potassium permanganate, but also because they themselves become changed, in fact if permanganate is powdered and then worked vigorously with such an excipient, reaction may be sufficiently violent to cause spontaneous combustion. It is necessary that the binding material shall be something which is not oxidised by permanganate of potash. Under ordinary conditions paraffin ointment, resin ointment, and anhydrous lanoline are such substances; a stiffening agent, however, is also necessary, and for this purpose Fuller's earth or kaolin is suitable. Liquorice and other vegetable powders are, of course, excluded. This is a convenient prescription:—

| | |
|---|---|
| ℞ Potassii Permanganatis ... ... ... | ʒss. |
| Kaolin ... ... ... ... ... ... | ʒj. |
| Adipis Lanæ... ... ... ... ... | ʒss. |

Misce. Fiant pilulæ xxx.

It has been recommended sometimes to omit the grease, and a mass can be made with 1½ grains of Fuller's earth to 1 grain of potassium permanganate, and a little water only.

Quinine is frequently given in pill form, both alone and with other ingredients. It does not as a rule present particular difficulty. It is desirable that a soluble salt should be prescribed, and when the sulphate is desired it is usual to add a little acid, which both assists the massing and gives a more soluble product. In the official pill of quinine sulphate 1 grain of tartaric acid is added to 30 grains of the sulphate, and a mass made with tragacanth and glycerin. It is by no means certain that this gives as soluble a pill as is obtained by using rather more tartaric acid—say 3 grains—omitting the tragacanth, and massing with glycerin and water in very small quantity. If it is desired to keep the size of quinine pills as small as possible, they may be made up with dilute sulphuric acid alone; a very small quantity suffices to yield a workable mass; in this case part of the quinine is converted into the bi-sulphate, a soluble salt.

Valerianate of zinc may be massed by means of glycerin of tragacanth after adding a little liquorice or marsh-mallow powder. Another method is to add a little gum acacia and mass with spirit.

(*To be continued.*)

